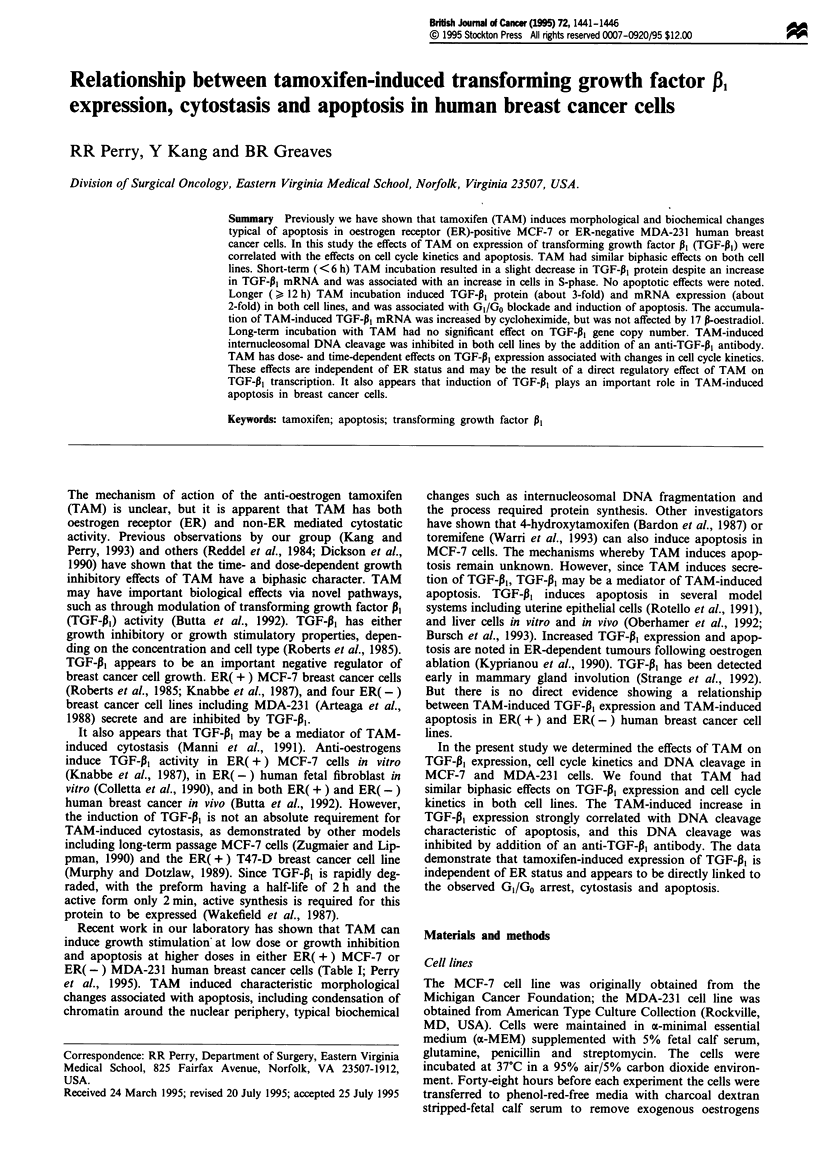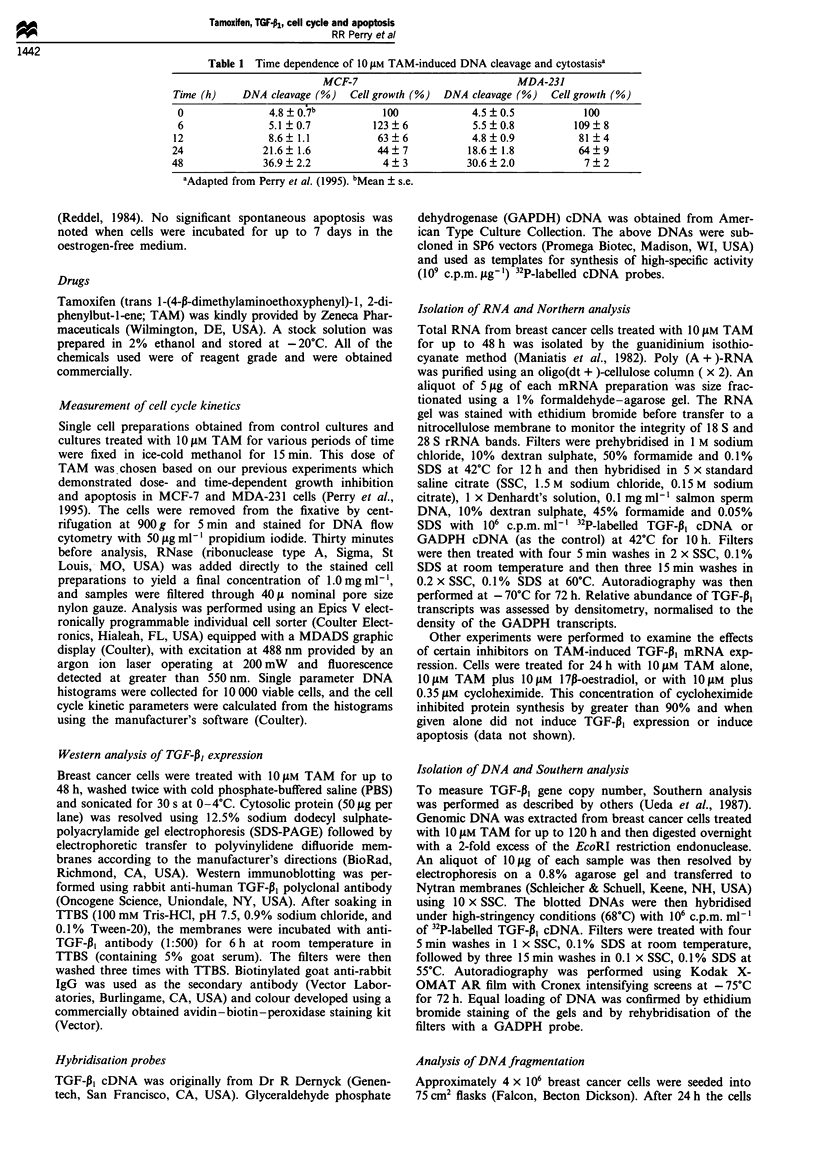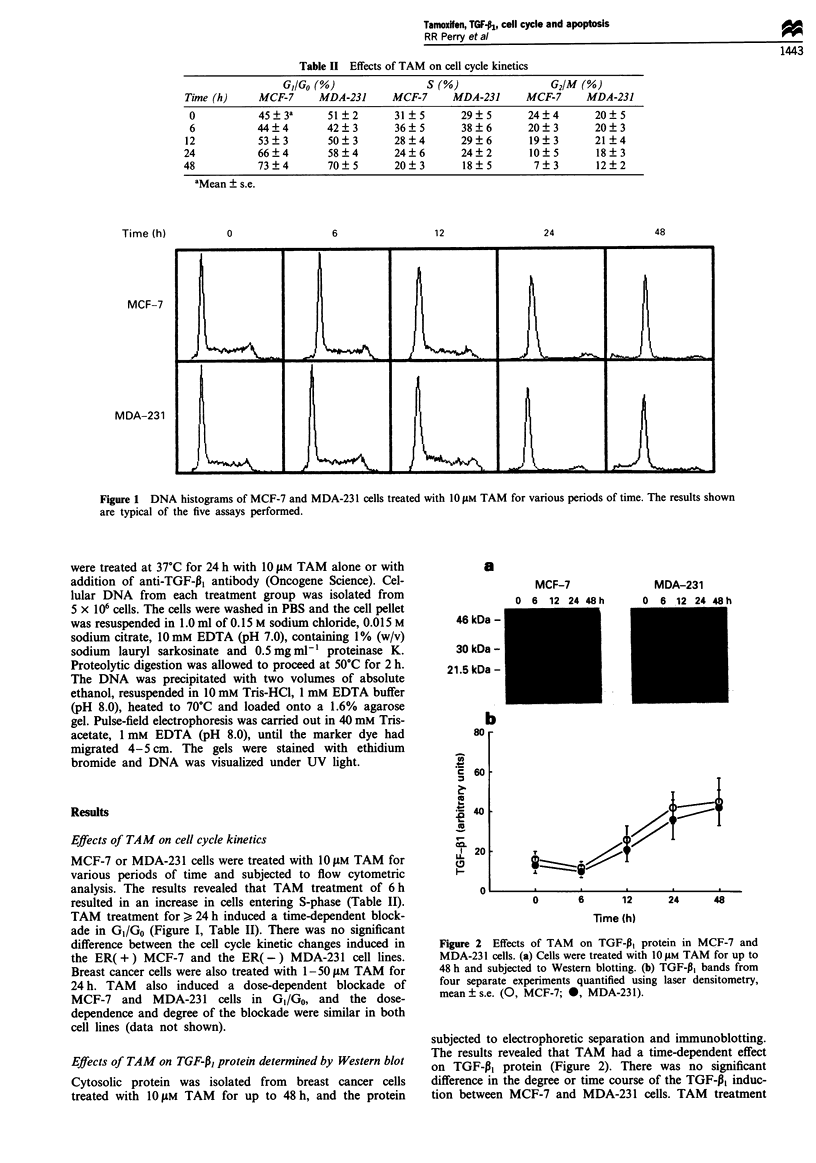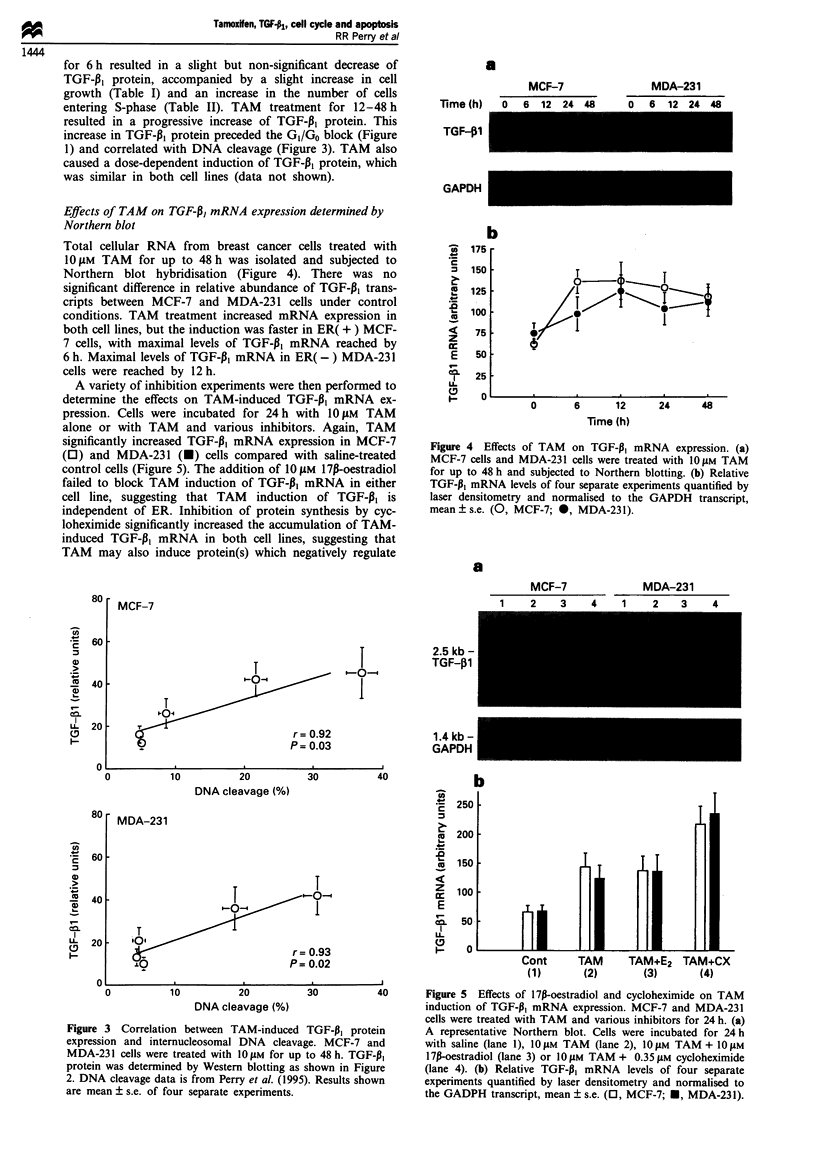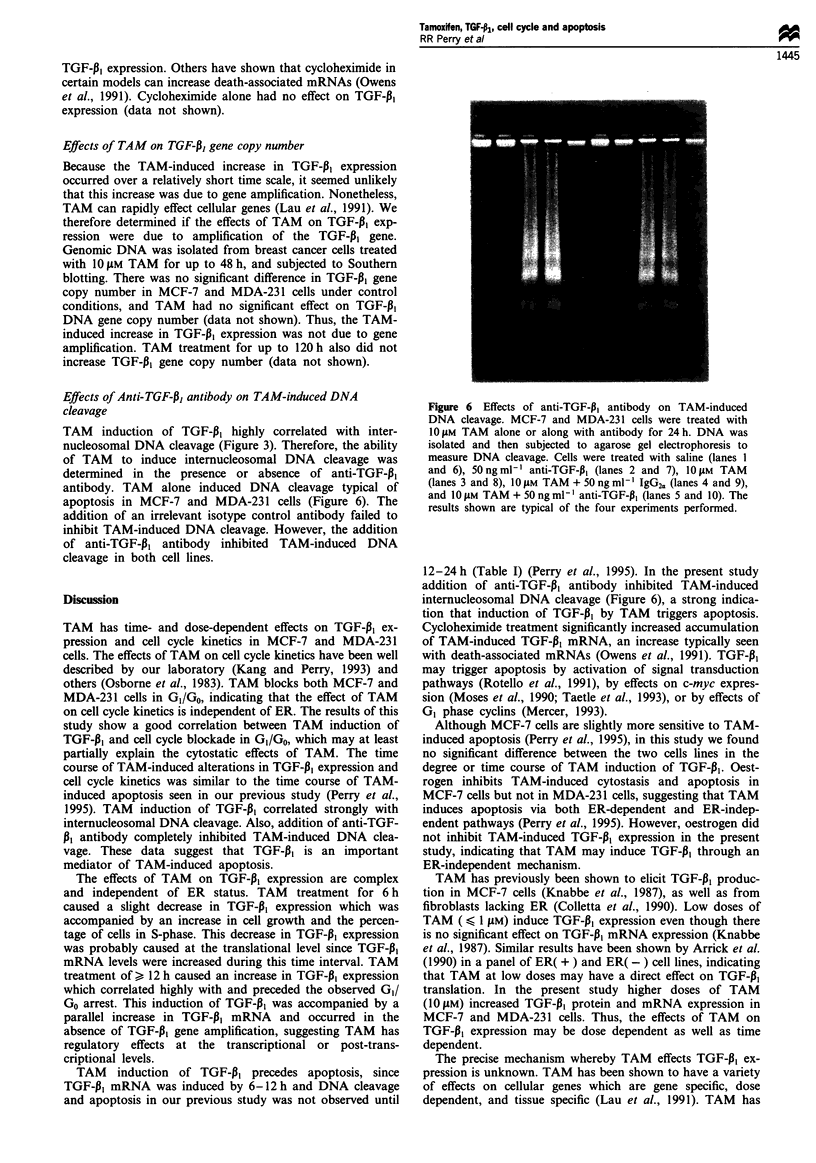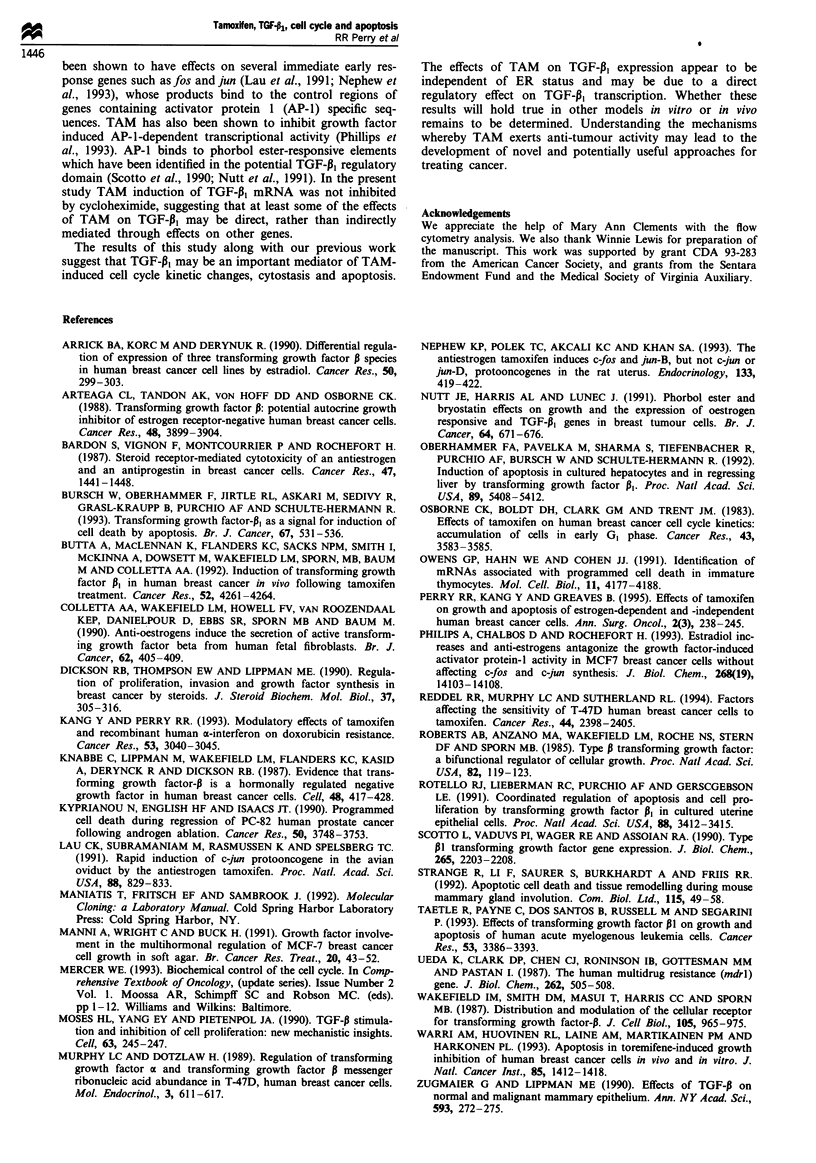# Relationship between tamoxifen-induced transforming growth factor beta 1 expression, cytostasis and apoptosis in human breast cancer cells.

**DOI:** 10.1038/bjc.1995.527

**Published:** 1995-12

**Authors:** R. R. Perry, Y. Kang, B. R. Greaves

**Affiliations:** Division of Surgical Oncology, Eastern Virginia Medical School, Norfolk 23507, USA.

## Abstract

**Images:**


British Journal of Cancer (1995) 72, 1441–1446
© 1995 Stockton Press All rights reserved 0007–0920/95 $12.00

### Relationship between tamoxifen-induced transforming growth factor $\beta_1$ expression, cytostasis and apoptosis in human breast cancer cells

RR Perry, Y Kang and BR Greaves

*Division of Surgical Oncology, Eastern Virginia Medical School, Norfolk, Virginia 23507, USA.*

**Summary** Previously we have shown that tamoxifen (TAM) induces morphological and biochemical changes typical of apoptosis in oestrogen receptor (ER)-positive MCF-7 or ER-negative MDA-231 human breast cancer cells. In this study the effects of TAM on expression of transforming growth factor $\beta_1$ (TGF-$\beta_1$) were correlated with the effects on cell cycle kinetics and apoptosis. TAM had similar biphasic effects on both cell lines. Short-term (<6 h) TAM incubation resulted in a slight decrease in TGF-$\beta_1$ protein despite an increase in TGF-$\beta_1$ mRNA and was associated with an increase in cells in S-phase. No apoptotic effects were noted. Longer ($\geqslant$ 12 h) TAM incubation induced TGF-$\beta_1$ protein (about 3-fold) and mRNA expression (about 2-fold) in both cell lines, and was associated with $G_1/G_0$ blockade and induction of apoptosis. The accumulation of TAM-induced TGF-$\beta_1$ mRNA was increased by cycloheximide, but was not affected by 17 $\beta$-oestradiol. Long-term incubation with TAM had no significant effect on TGF-$\beta_1$ gene copy number. TAM-induced internucleosomal DNA cleavage was inhibited in both cell lines by the addition of an anti-TGF-$\beta_1$ antibody. TAM has dose- and time-dependent effects on TGF-$\beta_1$ expression associated with changes in cell cycle kinetics. These effects are independent of ER status and may be the result of a direct regulatory effect of TAM on TGF-$\beta_1$ transcription. It also appears that induction of TGF-$\beta_1$ plays an important role in TAM-induced apoptosis in breast cancer cells.

**Keywords:** tamoxifen; apoptosis; transforming growth factor $\beta_1$

The mechanism of action of the anti-oestrogen tamoxifen (TAM) is unclear, but it is apparent that TAM has both oestrogen receptor (ER) and non-ER mediated cytostatic activity. Previous observations by our group (Kang and Perry, 1993) and others (Reddel *et al.*, 1984; Dickson *et al.*, 1990) have shown that the time- and dose-dependent growth inhibitory effects of TAM have a biphasic character. TAM may have important biological effects via novel pathways, such as through modulation of transforming growth factor $\beta_1$ (TGF-$\beta_1$) activity (Butta *et al.*, 1992). TGF-$\beta_1$ has either growth inhibitory or growth stimulatory properties, depending on the concentration and cell type (Roberts *et al.*, 1985). TGF-$\beta_1$ appears to be an important negative regulator of breast cancer cell growth. ER(+) MCF-7 breast cancer cells (Roberts *et al.*, 1985; Knabbe *et al.*, 1987), and four ER(−) breast cancer cell lines including MDA-231 (Arteaga *et al.*, 1988) secrete and are inhibited by TGF-$\beta_1$.

It also appears that TGF-$\beta_1$ may be a mediator of TAM-induced cytostasis (Manni *et al.*, 1991). Anti-oestrogens induce TGF-$\beta_1$ activity in ER(+) MCF-7 cells *in vitro* (Knabbe *et al.*, 1987), in ER(−) human fetal fibroblast *in vitro* (Colletta *et al.*, 1990), and in both ER(+) and ER(−) human breast cancer *in vivo* (Butta *et al.*, 1992). However, the induction of TGF-$\beta_1$ is not an absolute requirement for TAM-induced cytostasis, as demonstrated by other models including long-term passage MCF-7 cells (Zugmaier and Lippman, 1990) and the ER(+) T47-D breast cancer cell line (Murphy and Dotzlaw, 1989). Since TGF-$\beta_1$ is rapidly degraded, with the preform having a half-life of 2 h and the active form only 2 min, active synthesis is required for this protein to be expressed (Wakefield *et al.*, 1987).

Recent work in our laboratory has shown that TAM can induce growth stimulation at low dose or growth inhibition and apoptosis at higher doses in either ER(+) MCF-7 or ER(−) MDA-231 human breast cancer cells (Table I; Perry *et al.*, 1995). TAM induced characteristic morphological changes associated with apoptosis, including condensation of chromatin around the nuclear periphery, typical biochemical changes such as internucleosomal DNA fragmentation and the process required protein synthesis. Other investigators have shown that 4-hydroxytamoxifen (Bardon *et al.*, 1987) or toremifene (Warri *et al.*, 1993) can also induce apoptosis in MCF-7 cells. The mechanisms whereby TAM induces apoptosis remain unknown. However, since TAM induces secretion of TGF-$\beta_1$, TGF-$\beta_1$ may be a mediator of TAM-induced apoptosis. TGF-$\beta_1$ induces apoptosis in several model systems including uterine epithelial cells (Rotello *et al.*, 1991), and liver cells *in vitro* and *in vivo* (Oberhamer *et al.*, 1992; Bursch *et al.*, 1993). Increased TGF-$\beta_1$ expression and apoptosis are noted in ER-dependent tumours following oestrogen ablation (Kyprianou *et al.*, 1990). TGF-$\beta_1$ has been detected early in mammary gland involution (Strange *et al.*, 1992). But there is no direct evidence showing a relationship between TAM-induced TGF-$\beta_1$ expression and TAM-induced apoptosis in ER(+) and ER(−) human breast cancer cell lines.

In the present study we determined the effects of TAM on TGF-$\beta_1$ expression, cell cycle kinetics and DNA cleavage in MCF-7 and MDA-231 cells. We found that TAM had similar biphasic effects on TGF-$\beta_1$ expression and cell cycle kinetics in both cell lines. The TAM-induced increase in TGF-$\beta_1$ expression strongly correlated with DNA cleavage characteristic of apoptosis, and this DNA cleavage was inhibited by addition of an anti-TGF-$\beta_1$ antibody. The data demonstrate that tamoxifen-induced expression of TGF-$\beta_1$ is independent of ER status and appears to be directly linked to the observed $G_1/G_0$ arrest, cytostasis and apoptosis.

### Materials and methods

### *Cell lines*

The MCF-7 cell line was originally obtained from the Michigan Cancer Foundation; the MDA-231 cell line was obtained from American Type Culture Collection (Rockville, MD, USA). Cells were maintained in α-minimal essential medium (α-MEM) supplemented with 5% fetal calf serum, glutamine, penicillin and streptomycin. The cells were incubated at 37°C in a 95% air/5% carbon dioxide environment. Forty-eight hours before each experiment the cells were transferred to phenol-red-free media with charcoal dextran stripped-fetal calf serum to remove exogenous oestrogens

Correspondence: RR Perry, Department of Surgery, Eastern Virginia Medical School, 825 Fairfax Avenue, Norfolk, VA 23507-1912, USA.
Received 24 March 1995; revised 20 July 1995; accepted 25 July 1995

Table 1 Time dependence of 10 μM TAM-induced DNA cleavage and cytostasis[a]

| | MCF-7 | | MDA-231 | |
|---|---|---|---|---|
| *Time (h)* | *DNA cleavage (%)* | *Cell growth (%)* | *DNA cleavage (%)* | *Cell growth (%)* |
| 0 | 4.8 ± 0.7[b] | 100 | 4.5 ± 0.5 | 100 |
| 6 | 5.1 ± 0.7 | 123 ± 6 | 5.5 ± 0.8 | 109 ± 8 |
| 12 | 8.6 ± 1.1 | 63 ± 6 | 4.8 ± 0.9 | 81 ± 4 |
| 24 | 21.6 ± 1.6 | 44 ± 7 | 18.6 ± 1.8 | 64 ± 9 |
| 48 | 36.9 ± 2.2 | 4 ± 3 | 30.6 ± 2.0 | 7 ± 2 |

[a]Adapted from Perry *et al.* (1995). [b]Mean ± s.e.

(Reddel, 1984). No significant spontaneous apoptosis was noted when cells were incubated for up to 7 days in the oestrogen-free medium.

*Drugs*

Tamoxifen (trans 1-(4-β-dimethylaminoethoxyphenyl)-1, 2-diphenylbut-1-ene; TAM) was kindly provided by Zeneca Pharmaceuticals (Wilmington, DE, USA). A stock solution was prepared in 2% ethanol and stored at −20°C. All of the chemicals used were of reagent grade and were obtained commercially.

*Measurement of cell cycle kinetics*

Single cell preparations obtained from control cultures and cultures treated with 10 μM TAM for various periods of time were fixed in ice-cold methanol for 15 min. This dose of TAM was chosen based on our previous experiments which demonstrated dose- and time-dependent growth inhibition and apoptosis in MCF-7 and MDA-231 cells (Perry *et al.*, 1995). The cells were removed from the fixative by centrifugation at 900 *g* for 5 min and stained for DNA flow cytometry with 50 μg $ml^{-1}$ propidium iodide. Thirty minutes before analysis, RNase (ribonuclease type A, Sigma, St Louis, MO, USA) was added directly to the stained cell preparations to yield a final concentration of 1.0 mg $ml^{-1}$, and samples were filtered through 40 μ nominal pore size nylon gauze. Analysis was performed using an Epics V electronically programmable individual cell sorter (Coulter Electronics, Hialeah, FL, USA) equipped with a MDADS graphic display (Coulter), with excitation at 488 nm provided by an argon ion laser operating at 200 mW and fluorescence detected at greater than 550 nm. Single parameter DNA histograms were collected for 10 000 viable cells, and the cell cycle kinetic parameters were calculated from the histograms using the manufacturer's software (Coulter).

*Western analysis of TGF-$\beta_1$ expression*

Breast cancer cells were treated with 10 μM TAM for up to 48 h, washed twice with cold phosphate-buffered saline (PBS) and sonicated for 30 s at 0–4°C. Cytosolic protein (50 μg per lane) was resolved using 12.5% sodium dodecyl sulphate-polyacrylamide gel electrophoresis (SDS-PAGE) followed by electrophoretic transfer to polyvinylidene difluoride membranes according to the manufacturer's directions (BioRad, Richmond, CA, USA). Western immunoblotting was performed using rabbit anti-human TGF-$\beta_1$ polyclonal antibody (Oncogene Science, Uniondale, NY, USA). After soaking in TTBS (100 mM Tris-HCl, pH 7.5, 0.9% sodium chloride, and 0.1% Tween-20), the membranes were incubated with anti-TGF-$\beta_1$ antibody (1:500) for 6 h at room temperature in TTBS (containing 5% goat serum). The filters were then washed three times with TTBS. Biotinylated goat anti-rabbit IgG was used as the secondary antibody (Vector Laboratories, Burlingame, CA, USA) and colour developed using a commercially obtained avidin–biotin–peroxidase staining kit (Vector).

*Hybridisation probes*

TGF-$\beta_1$ cDNA was originally from Dr R Dernyck (Genentech, San Francisco, CA, USA). Glyceraldehyde phosphate dehydrogenase (GAPDH) cDNA was obtained from American Type Culture Collection. The above DNAs were subcloned in SP6 vectors (Promega Biotec, Madison, WI, USA) and used as templates for synthesis of high-specific activity ($10^9$ c.p.m. $\mu g^{-1}$) $^{32}P$-labelled cDNA probes.

*Isolation of RNA and Northern analysis*

Total RNA from breast cancer cells treated with 10 μM TAM for up to 48 h was isolated by the guanidinium isothiocyanate method (Maniatis *et al.*, 1982). Poly (A + )-RNA was purified using an oligo(dt + )-cellulose column ( × 2). An aliquot of 5 μg of each mRNA preparation was size fractionated using a 1% formaldehyde–agarose gel. The RNA gel was stained with ethidium bromide before transfer to a nitrocellulose membrane to monitor the integrity of 18 S and 28 S rRNA bands. Filters were prehybridised in 1 M sodium chloride, 10% dextran sulphate, 50% formamide and 0.1% SDS at 42°C for 12 h and then hybridised in 5 × standard saline citrate (SSC, 1.5 M sodium chloride, 0.15 M sodium citrate), 1 × Denhardt's solution, 0.1 mg $ml^{-1}$ salmon sperm DNA, 10% dextran sulphate, 45% formamide and 0.05% SDS with $10^6$ c.p.m. $ml^{-1}$ $^{32}P$-labelled TGF-$\beta_1$ cDNA or GADPH cDNA (as the control) at 42°C for 10 h. Filters were then treated with four 5 min washes in 2 × SSC, 0.1% SDS at room temperature and then three 15 min washes in 0.2 × SSC, 0.1% SDS at 60°C. Autoradiography was then performed at −70°C for 72 h. Relative abundance of TGF-$\beta_1$ transcripts was assessed by densitometry, normalised to the density of the GADPH transcripts.

Other experiments were performed to examine the effects of certain inhibitors on TAM-induced TGF-$\beta_1$ mRNA expression. Cells were treated for 24 h with 10 μM TAM alone, 10 μM TAM plus 10 μM 17β-oestradiol, or with 10 μM plus 0.35 μM cycloheximide. This concentration of cycloheximide inhibited protein synthesis by greater than 90% and when given alone did not induce TGF-$\beta_1$ expression or induce apoptosis (data not shown).

*Isolation of DNA and Southern analysis*

To measure TGF-$\beta_1$ gene copy number, Southern analysis was performed as described by others (Ueda *et al.*, 1987). Genomic DNA was extracted from breast cancer cells treated with 10 μM TAM for up to 120 h and then digested overnight with a 2-fold excess of the *Eco*RI restriction endonuclease. An aliquot of 10 μg of each sample was then resolved by electrophoresis on a 0.8% agarose gel and transferred to Nytran membranes (Schleicher & Schuell, Keene, NH, USA) using 10 × SSC. The blotted DNAs were then hybridised under high-stringency conditions (68°C) with $10^6$ c.p.m. $ml^{-1}$ of $^{32}P$-labelled TGF-$\beta_1$ cDNA. Filters were treated with four 5 min washes in 1 × SSC, 0.1% SDS at room temperature, followed by three 15 min washes in 0.1 × SSC, 0.1% SDS at 55°C. Autoradiography was performed using Kodak X-OMAT AR film with Cronex intensifying screens at −75°C for 72 h. Equal loading of DNA was confirmed by ethidium bromide staining of the gels and by rehybridisation of the filters with a GADPH probe.

*Analysis of DNA fragmentation*

Approximately 4 × $10^6$ breast cancer cells were seeded into 75 $cm^2$ flasks (Falcon, Becton Dickson). After 24 h the cells

**Table II** Effects of TAM on cell cycle kinetics

| | $G_1/G_0$ (%) | | S (%) | | $G_2/M$ (%) | |
|---|---|---|---|---|---|---|
| *Time (h)* | *MCF-7* | *MDA-231* | *MCF-7* | *MDA-231* | *MCF-7* | *MDA-231* |
| 0 | 45 ± 3[a] | 51 ± 2 | 31 ± 5 | 29 ± 5 | 24 ± 4 | 20 ± 5 |
| 6 | 44 ± 4 | 42 ± 3 | 36 ± 5 | 38 ± 6 | 20 ± 3 | 20 ± 3 |
| 12 | 53 ± 3 | 50 ± 3 | 28 ± 4 | 29 ± 6 | 19 ± 3 | 21 ± 4 |
| 24 | 66 ± 4 | 58 ± 4 | 24 ± 6 | 24 ± 2 | 10 ± 5 | 18 ± 3 |
| 48 | 73 ± 4 | 70 ± 5 | 20 ± 3 | 18 ± 5 | 7 ± 3 | 12 ± 2 |

[a]Mean ± s.e.

**Figure 1** DNA histograms of MCF-7 and MDA-231 cells treated with 10 μM TAM for various periods of time. The results shown are typical of the five assays performed.

were treated at 37°C for 24 h with 10 μM TAM alone or with addition of anti-TGF-$\beta_1$ antibody (Oncogene Science). Cellular DNA from each treatment group was isolated from $5 \times 10^6$ cells. The cells were washed in PBS and the cell pellet was resuspended in 1.0 ml of 0.15 M sodium chloride, 0.015 M sodium citrate, 10 mM EDTA (pH 7.0), containing 1% (w/v) sodium lauryl sarkosinate and 0.5 mg $ml^{-1}$ proteinase K. Proteolytic digestion was allowed to proceed at 50°C for 2 h. The DNA was precipitated with two volumes of absolute ethanol, resuspended in 10 mM Tris-HCl, 1 mM EDTA buffer (pH 8.0), heated to 70°C and loaded onto a 1.6% agarose gel. Pulse-field electrophoresis was carried out in 40 mM Tris-acetate, 1 mM EDTA (pH 8.0), until the marker dye had migrated 4–5 cm. The gels were stained with ethidium bromide and DNA was visualized under UV light.

### Results

### *Effects of TAM on cell cycle kinetics*

MCF-7 or MDA-231 cells were treated with 10 μM TAM for various periods of time and subjected to flow cytometric analysis. The results revealed that TAM treatment of 6 h resulted in an increase in cells entering S-phase (Table II). TAM treatment for ⩾ 24 h induced a time-dependent blockade in $G_1/G_0$ (Figure I, Table II). There was no significant difference between the cell cycle kinetic changes induced in the ER( + ) MCF-7 and the ER( – ) MDA-231 cell lines. Breast cancer cells were also treated with 1–50 μM TAM for 24 h. TAM also induced a dose-dependent blockade of MCF-7 and MDA-231 cells in $G_1/G_0$, and the dose-dependence and degree of the blockade were similar in both cell lines (data not shown).

### *Effects of TAM on TGF-$\beta_1$ protein determined by Western blot*

Cytosolic protein was isolated from breast cancer cells treated with 10 μM TAM for up to 48 h, and the protein

**Figure 2** Effects of TAM on TGF-$\beta_1$ protein in MCF-7 and MDA-231 cells. (**a**) Cells were treated with 10 μM TAM for up to 48 h and subjected to Western blotting. (**b**) TGF-$\beta_1$ bands from four separate experiments quantified using laser densitometry, mean ± s.e. (O, MCF-7; ●, MDA-231).

subjected to electrophoretic separation and immunoblotting. The results revealed that TAM had a time-dependent effect on TGF-$\beta_1$ protein (Figure 2). There was no significant difference in the degree or time course of the TGF-$\beta_1$ induction between MCF-7 and MDA-231 cells. TAM treatment for 6 h resulted in a slight but non-significant decrease of TGF-$\beta_1$ protein, accompanied by a slight increase in cell growth (Table I) and an increase in the number of cells entering S-phase (Table II). TAM treatment for 12–48 h resulted in a progressive increase of TGF-$\beta_1$ protein. This increase in TGF-$\beta_1$ protein preceded the $G_1/G_0$ block (Figure 1) and correlated with DNA cleavage (Figure 3). TAM also caused a dose-dependent induction of TGF-$\beta_1$ protein, which was similar in both cell lines (data not shown).

*Effects of TAM on TGF-$\beta_1$ mRNA expression determined by Northern blot*

Total cellular RNA from breast cancer cells treated with 10 μM TAM for up to 48 h was isolated and subjected to Northern blot hybridisation (Figure 4). There was no significant difference in relative abundance of TGF-$\beta_1$ transcripts between MCF-7 and MDA-231 cells under control conditions. TAM treatment increased mRNA expression in both cell lines, but the induction was faster in ER(+) MCF-7 cells, with maximal levels of TGF-$\beta_1$ mRNA reached by 6 h. Maximal levels of TGF-$\beta_1$ mRNA in ER(−) MDA-231 cells were reached by 12 h.

A variety of inhibition experiments were then performed to determine the effects on TAM-induced TGF-$\beta_1$ mRNA expression. Cells were incubated for 24 h with 10 μM TAM alone or with TAM and various inhibitors. Again, TAM significantly increased TGF-$\beta_1$ mRNA expression in MCF-7 (□) and MDA-231 (■) cells compared with saline-treated control cells (Figure 5). The addition of 10 μM 17$\beta$-oestradiol failed to block TAM induction of TGF-$\beta_1$ mRNA in either cell line, suggesting that TAM induction of TGF-$\beta_1$ is independent of ER. Inhibition of protein synthesis by cycloheximide significantly increased the accumulation of TAM-induced TGF-$\beta_1$ mRNA in both cell lines, suggesting that TAM may also induce protein(s) which negatively regulate

**Figure 3** Correlation between TAM-induced TGF-$\beta_1$ protein expression and internucleosomal DNA cleavage. MCF-7 and MDA-231 cells were treated with 10 μM for up to 48 h. TGF-$\beta_1$ protein was determined by Western blotting as shown in Figure 2. DNA cleavage data is from Perry *et al.* (1995). Results shown are mean ± s.e. of four separate experiments.

**Figure 4** Effects of TAM on TGF-$\beta_1$ mRNA expression. (**a**) MCF-7 cells and MDA-231 cells were treated with 10 μM TAM for up to 48 h and subjected to Northern blotting. (**b**) Relative TGF-$\beta_1$ mRNA levels of four separate experiments quantified by laser densitometry and normalised to the GAPDH transcript, mean ± s.e. (○, MCF-7; ●, MDA-231).

**Figure 5** Effects of 17$\beta$-oestradiol and cycloheximide on TAM induction of TGF-$\beta_1$ mRNA expression. MCF-7 and MDA-231 cells were treated with TAM and various inhibitors for 24 h. (**a**) A representative Northern blot. Cells were incubated for 24 h with saline (lane 1), 10 μM TAM (lane 2), 10 μM TAM + 10 μM 17$\beta$-oestradiol (lane 3) or 10 μM TAM + 0.35 μM cycloheximide (lane 4). (**b**) Relative TGF-$\beta_1$ mRNA levels of four separate experiments quantified by laser densitometry and normalised to the GADPH transcript, mean ± s.e. (□, MCF-7; ■, MDA-231).

TGF-$\beta_1$ expression. Others have shown that cycloheximide in certain models can increase death-associated mRNAs (Owens *et al.*, 1991). Cycloheximide alone had no effect on TGF-$\beta_1$ expression (data not shown).

### *Effects of TAM on TGF-$\beta_1$ gene copy number*

Because the TAM-induced increase in TGF-$\beta_1$ expression occurred over a relatively short time scale, it seemed unlikely that this increase was due to gene amplification. Nonetheless, TAM can rapidly effect cellular genes (Lau *et al.*, 1991). We therefore determined if the effects of TAM on TGF-$\beta_1$ expression were due to amplification of the TGF-$\beta_1$ gene. Genomic DNA was isolated from breast cancer cells treated with 10 $\mu$M TAM for up to 48 h, and subjected to Southern blotting. There was no significant difference in TGF-$\beta_1$ gene copy number in MCF-7 and MDA-231 cells under control conditions, and TAM had no significant effect on TGF-$\beta_1$ DNA gene copy number (data not shown). Thus, the TAM-induced increase in TGF-$\beta_1$ expression was not due to gene amplification. TAM treatment for up to 120 h also did not increase TGF-$\beta_1$ gene copy number (data not shown).

### *Effects of Anti-TGF-$\beta_1$ antibody on TAM-induced DNA cleavage*

TAM induction of TGF-$\beta_1$ highly correlated with internucleosomal DNA cleavage (Figure 3). Therefore, the ability of TAM to induce internucleosomal DNA cleavage was determined in the presence or absence of anti-TGF-$\beta_1$ antibody. TAM alone induced DNA cleavage typical of apoptosis in MCF-7 and MDA-231 cells (Figure 6). The addition of an irrelevant isotype control antibody failed to inhibit TAM-induced DNA cleavage. However, the addition of anti-TGF-$\beta_1$ antibody inhibited TAM-induced DNA cleavage in both cell lines.

**Figure 6** Effects of anti-TGF-$\beta_1$ antibody on TAM-induced DNA cleavage. MCF-7 and MDA-231 cells were treated with 10 $\mu$M TAM alone or along with antibody for 24 h. DNA was isolated and then subjected to agarose gel electrophoresis to measure DNA cleavage. Cells were treated with saline (lanes 1 and 6), 50 ng ml$^{-1}$ anti-TGF-$\beta_1$ (lanes 2 and 7), 10 $\mu$M TAM (lanes 3 and 8), 10 $\mu$M TAM + 50 ng ml$^{-1}$ Ig$G_{2\alpha}$ (lanes 4 and 9), and 10 $\mu$M TAM + 50 ng ml$^{-1}$ anti-TGF-$\beta_1$ (lanes 5 and 10). The results shown are typical of the four experiments performed.

### Discussion

TAM has time- and dose-dependent effects on TGF-$\beta_1$ expression and cell cycle kinetics in MCF-7 and MDA-231 cells. The effects of TAM on cell cycle kinetics have been well described by our laboratory (Kang and Perry, 1993) and others (Osborne *et al.*, 1983). TAM blocks both MCF-7 and MDA-231 cells in $G_1/G_0$, indicating that the effect of TAM on cell cycle kinetics is independent of ER. The results of this study show a good correlation between TAM induction of TGF-$\beta_1$ and cell cycle blockade in $G_1/G_0$, which may at least partially explain the cytostatic effects of TAM. The time course of TAM-induced alterations in TGF-$\beta_1$ expression and cell cycle kinetics was similar to the time course of TAM-induced apoptosis seen in our previous study (Perry *et al.*, 1995). TAM induction of TGF-$\beta_1$ correlated strongly with internucleosomal DNA cleavage. Also, addition of anti-TGF-$\beta_1$ antibody completely inhibited TAM-induced DNA cleavage. These data suggest that TGF-$\beta_1$ is an important mediator of TAM-induced apoptosis.

The effects of TAM on TGF-$\beta_1$ expression are complex and independent of ER status. TAM treatment for 6 h caused a slight decrease in TGF-$\beta_1$ expression which was accompanied by an increase in cell growth and the percentage of cells in S-phase. This decrease in TGF-$\beta_1$ expression was probably caused at the translational level since TGF-$\beta_1$ mRNA levels were increased during this time interval. TAM treatment of $\geqslant$ 12 h caused an increase in TGF-$\beta_1$ expression which correlated highly with and preceded the observed $G_1/G_0$ arrest. This induction of TGF-$\beta_1$ was accompanied by a parallel increase in TGF-$\beta_1$ mRNA and occurred in the absence of TGF-$\beta_1$ gene amplification, suggesting TAM has regulatory effects at the transcriptional or post-transcriptional levels.

TAM induction of TGF-$\beta_1$ precedes apoptosis, since TGF-$\beta_1$ mRNA was induced by 6–12 h and DNA cleavage and apoptosis in our previous study was not observed until 12–24 h (Table I) (Perry *et al.*, 1995). In the present study addition of anti-TGF-$\beta_1$ antibody inhibited TAM-induced internucleosomal DNA cleavage (Figure 6), a strong indication that induction of TGF-$\beta_1$ by TAM triggers apoptosis. Cycloheximide treatment significantly increased accumulation of TAM-induced TGF-$\beta_1$ mRNA, an increase typically seen with death-associated mRNAs (Owens *et al.*, 1991). TGF-$\beta_1$ may trigger apoptosis by activation of signal transduction pathways (Rotello *et al.*, 1991), by effects on c-*myc* expression (Moses *et al.*, 1990; Taetle *et al.*, 1993), or by effects of $G_1$ phase cyclins (Mercer, 1993).

Although MCF-7 cells are slightly more sensitive to TAM-induced apoptosis (Perry *et al.*, 1995), in this study we found no significant difference between the two cells lines in the degree or time course of TAM induction of TGF-$\beta_1$. Oestrogen inhibits TAM-induced cytostasis and apoptosis in MCF-7 cells but not in MDA-231 cells, suggesting that TAM induces apoptosis via both ER-dependent and ER-independent pathways (Perry *et al.*, 1995). However, oestrogen did not inhibit TAM-induced TGF-$\beta_1$ expression in the present study, indicating that TAM may induce TGF-$\beta_1$ through an ER-independent mechanism.

TAM has previously been shown to elicit TGF-$\beta_1$ production in MCF-7 cells (Knabbe *et al.*, 1987), as well as from fibroblasts lacking ER (Colletta *et al.*, 1990). Low doses of TAM ($\leqslant$ 1 $\mu$M) induce TGF-$\beta_1$ expression even though there is no significant effect on TGF-$\beta_1$ mRNA expression (Knabbe *et al.*, 1987). Similar results have been shown by Arrick *et al.* (1990) in a panel of ER( + ) and ER( − ) cell lines, indicating that TAM at low doses may have a direct effect on TGF-$\beta_1$ translation. In the present study higher doses of TAM (10 $\mu$M) increased TGF-$\beta_1$ protein and mRNA expression in MCF-7 and MDA-231 cells. Thus, the effects of TAM on TGF-$\beta_1$ expression may be dose dependent as well as time dependent.

The precise mechanism whereby TAM effects TGF-$\beta_1$ expression is unknown. TAM has been shown to have a variety of effects on cellular genes which are gene specific, dose dependent, and tissue specific (Lau *et al.*, 1991). TAM has been shown to have effects on several immediate early response genes such as *fos* and *jun* (Lau *et al.*, 1991; Nephew *et al.*, 1993), whose products bind to the control regions of genes containing activator protein 1 (AP-1) specific sequences. TAM has also been shown to inhibit growth factor induced AP-1-dependent transcriptional activity (Phillips *et al.*, 1993). AP-1 binds to phorbol ester-responsive elements which have been identified in the potential TGF-$\beta_1$ regulatory domain (Scotto *et al.*, 1990; Nutt *et al.*, 1991). In the present study TAM induction of TGF-$\beta_1$ mRNA was not inhibited by cycloheximide, suggesting that at least some of the effects of TAM on TGF-$\beta_1$ may be direct, rather than indirectly mediated through effects on other genes.

The results of this study along with our previous work suggest that TGF-$\beta_1$ may be an important mediator of TAM-induced cell cycle kinetic changes, cytostasis and apoptosis. The effects of TAM on TGF-$\beta_1$ expression appear to be independent of ER status and may be due to a direct regulatory effect on TGF-$\beta_1$ transcription. Whether these results will hold true in other models *in vitro* or *in vivo* remains to be determined. Understanding the mechanisms whereby TAM exerts anti-tumour activity may lead to the development of novel and potentially useful approaches for treating cancer.

**Acknowledgements**
We appreciate the help of Mary Ann Clements with the flow cytometry analysis. We also thank Winnie Lewis for preparation of the manuscript. This work was supported by grant CDA 93-283 from the American Cancer Society, and grants from the Sentara Endowment Fund and the Medical Society of Virginia Auxiliary.

**References**

ARRICK BA, KORC M AND DERYNUK R. (1990). Differential regulation of expression of three transforming growth factor $\beta$ species in human breast cancer cell lines by estradiol. *Cancer Res.*, **50,** 299–303.

ARTEAGA CL, TANDON AK, VON HOFF DD AND OSBORNE CK. (1988). Transforming growth factor $\beta$: potential autocrine growth inhibitor of estrogen receptor-negative human breast cancer cells. *Cancer Res.*, **48,** 3899–3904.

BARDON S, VIGNON F, MONTCOURRIER P AND ROCHEFORT H. (1987). Steroid receptor-mediated cytotoxicity of an antiestrogen and an antiprogestin in breast cancer cells. *Cancer Res.*, **47,** 1441–1448.

BURSCH W, OBERHAMMER F, JIRTLE RL, ASKARI M, SEDIVY R, GRASL-KRAUPP B, PURCHIO AF AND SCHULTE-HERMANN R. (1993). Transforming growth factor-$\beta_1$ as a signal for induction of cell death by apoptosis. *Br. J. Cancer*, **67,** 531–536.

BUTTA A, MACLENNAN K, FLANDERS KC, SACKS NPM, SMITH I, MCKINNA A, DOWSETT M, WAKEFIELD LM, SPORN, MB, BAUM M AND COLLETTA AA. (1992). Induction of transforming growth factor $\beta_1$ in human breast cancer *in vivo* following tamoxifen treatment. *Cancer Res.*, **52,** 4261–4264.

COLLETTA AA, WAKEFIELD LM, HOWELL FV, VAN ROOZENDAAL KEP, DANIELPOUR D, EBBS SR, SPORN MB AND BAUM M. (1990). Anti-oestrogens induce the secretion of active transforming growth factor beta from human fetal fibroblasts. *Br. J. Cancer*, **62,** 405–409.

DICKSON RB, THOMPSON EW AND LIPPMAN ME. (1990). Regulation of proliferation, invasion and growth factor synthesis in breast cancer by steroids. *J. Steroid Biochem. Mol. Biol.*, **37,** 305–316.

KANG Y AND PERRY RR. (1993). Modulatory effects of tamoxifen and recombinant human $\alpha$-interferon on doxorubicin resistance. *Cancer Res.*, **53,** 3040–3045.

KNABBE C, LIPPMAN M, WAKEFIELD LM, FLANDERS KC, KASID A, DERYNCK R AND DICKSON RB. (1987). Evidence that transforming growth factor-$\beta$ is a hormonally regulated negative growth factor in human breast cancer cells. *Cell*, **48,** 417–428.

KYPRIANOU N, ENGLISH HF AND ISAACS JT. (1990). Programmed cell death during regression of PC-82 human prostate cancer following androgen ablation. *Cancer Res.*, **50,** 3748–3753.

LAU CK, SUBRAMANIAM M, RASMUSSEN K AND SPELSBERG TC. (1991). Rapid induction of c-*jun* protooncogene in the avian oviduct by the antiestrogen tamoxifen. *Proc. Natl. Acad. Sci. USA*, **88,** 829–833.

MANIATIS T, FRITSCH EF AND SAMBROOK J. (1992). *Molecular Cloning: a Laboratory Manual.* Cold Spring Harbor Laboratory Press: Cold Spring Harbor, NY.

MANNI A, WRIGHT C AND BUCK H. (1991). Growth factor involvement in the multihormonal regulation of MCF-7 breast cancer cell growth in soft agar. *Br. Cancer Res. Treat.*, **20,** 43–52.

MERCER WE. (1993). Biochemical control of the cell cycle. In *Comprehensive Textbook of Oncology*, (update series). Issue Number 2 Vol. 1. Moossa AR, Schimpff SC and Robson MC. (eds). pp 1–12. Williams and Wilkins: Baltimore.

MOSES HL, YANG EY AND PIETENPOL JA. (1990). TGF-$\beta$ stimulation and inhibition of cell proliferation: new mechanistic insights. *Cell*, **63,** 245–247.

MURPHY LC AND DOTZLAW H. (1989). Regulation of transforming growth factor $\alpha$ and transforming growth factor $\beta$ messenger ribonucleic acid abundance in T-47D, human breast cancer cells. *Mol. Endocrinol.*, **3,** 611–617.

NEPHEW KP, POLEK TC, AKCALI KC AND KHAN SA. (1993). The antiestrogen tamoxifen induces c-*fos* and *jun*-B, but not c-*jun* or *jun*-D, protooncogenes in the rat uterus. *Endocrinology*, **133,** 419–422.

NUTT JE, HARRIS AL AND LUNEC J. (1991). Phorbol ester and bryostatin effects on growth and the expression of oestrogen responsive and TGF-$\beta_1$ genes in breast tumour cells. *Br. J. Cancer*, **64,** 671–676.

OBERHAMMER FA, PAVELKA M, SHARMA S, TIEFENBACHER R, PURCHIO AF, BURSCH W AND SCHULTE-HERMANN R. (1992). Induction of apoptosis in cultured hepatocytes and in regressing liver by transforming growth factor $\beta_1$. *Proc. Natl Acad. Sci. USA*, **89,** 5408–5412.

OSBORNE CK, BOLDT DH, CLARK GM AND TRENT JM. (1983). Effects of tamoxifen on human breast cancer cell cycle kinetics: accumulation of cells in early $G_1$ phase. *Cancer Res.*, **43,** 3583–3585.

OWENS GP, HAHN WE AND COHEN JJ. (1991). Identification of mRNAs associated with programmed cell death in immature thymocytes. *Mol. Cell. Biol.*, **11,** 4177–4188.

PERRY RR, KANG Y AND GREAVES B. (1995). Effects of tamoxifen on growth and apoptosis of estrogen-dependent and -independent human breast cancer cells. *Ann. Surg. Oncol.*, **2(3),** 238–245.

PHILIPS A, CHALBOS D AND ROCHEFORT H. (1993). Estradiol increases and anti-estrogens antagonize the growth factor-induced activator protein-1 activity in MCF7 breast cancer cells without affecting c-*fos* and c-*jun* synthesis. *J. Biol. Chem.*, **268(19),** 14103–14108.

REDDEL RR, MURPHY LC AND SUTHERLAND RL. (1994). Factors affecting the sensitivity of T-47D human breast cancer cells to tamoxifen. *Cancer Res.*, **44,** 2398–2405.

ROBERTS AB, ANZANO MA, WAKEFIELD LM, ROCHE NS, STERN DF AND SPORN MB. (1985). Type $\beta$ transforming growth factor: a bifunctional regulator of cellular growth. *Proc. Natl Acad. Sci. USA*, **82,** 119–123.

ROTELLO RJ, LIEBERMAN RC, PURCHIO AF AND GERSCGEBSON LE. (1991). Coordinated regulation of apoptosis and cell proliferation by transforming growth factor $\beta_1$ in cultured uterine epithelial cells. *Proc. Natl Acad. Sci. USA*, **88,** 3412–3415.

SCOTTO L, VADUVS PI, WAGER RE AND ASSOIAN RA. (1990). Type $\beta 1$ transforming growth factor gene expression. *J. Biol. Chem.*, **265,** 2203–2208.

STRANGE R, LI F, SAURER S, BURKHARDT A AND FRIIS RR. (1992). Apoptotic cell death and tissue remodelling during mouse mammary gland involution. *Com. Biol. Ltd.*, **115,** 49–58.

TAETLE R, PAYNE C, DOS SANTOS B, RUSSELL M AND SEGARINI P. (1993). Effects of transforming growth factor $\beta 1$ on growth and apoptosis of human acute myelogenous leukemia cells. *Cancer Res.*, **53,** 3386–3393.

UEDA K, CLARK DP, CHEN CJ, RONINSON IB, GOTTESMAN MM AND PASTAN I. (1987). The human multidrug resistance (*mdr*1) gene. *J. Biol. Chem.*, **262,** 505–508.

WAKEFIELD IM, SMITH DM, MASUI T, HARRIS CC AND SPORN MB. (1987). Distribution and modulation of the cellular receptor for transforming growth factor-$\beta$. *J. Cell Biol.*, **105,** 965–975.

WARRI AM, HUOVINEN RL, LAINE AM, MARTIKAINEN PM AND HARKONEN PL. (1993). Apoptosis in toremifene-induced growth inhibition of human breast cancer cells *in vivo* and *in vitro*. *J. Natl. Cancer Inst.*, **85,** 1412–1418.

ZUGMAIER G AND LIPPMAN ME (1990). Effects of TGF-$\beta$ on normal and malignant mammary epithelium. *Ann. NY Acad. Sci.*, **593,** 272–275.